# A Thermal Flow Sensor Based on Printed Circuit Technology in Constant Temperature Mode for Various Fluids

**DOI:** 10.3390/s19051065

**Published:** 2019-03-02

**Authors:** Thomas Glatzl, Roman Beigelbeck, Samir Cerimovic, Harald Steiner, Florian Wenig, Thilo Sauter, Albert Treytl, Franz Keplinger

**Affiliations:** 1 Department for Integrated Sensor Systems, Danube University Krems, Viktor Kaplan Strasse 2 E, 2700 Wiener Neustadt, Austria; roman.beigelbeck@donau-uni.ac.at (R.B.); samir.cerimovic@donau-uni.ac.at (S.C.); harald.steiner@donau-uni.ac.at (H.S.); thilo.sauter@donau-uni.ac.at (T.S.); Albert.treytl@donau-uni.ac.at (A.T.); 2 Center for Building Technology, University of Applied Sciences Burgenland, Steinamangerstrasse 21, 7423 Pinkafeld, Austria; florian.wenig@forschung-burgenland.at; 3 Institute of Sensor and Actuator Systems, TU Wien, Gusshausstrasse 27-29, 1040 Vienna, Austria; franz.keplinger@tuwien.ac.at

**Keywords:** thermal flow sensor, calorimetric, CT mode, printed circuit board, HVAC systems

## Abstract

We present a thermal flow sensor designed for measuring air as well as water flow velocities in heating, ventilation, and air conditioning (HVAC) systems. The sensor is designed to integrate the flow along the entire diameter of the pipe also quantifying the volume flow rate of the streaming fluid where the calorimetric principle in constant temperature operation is utilized as a readout method. In the constant temperature mode, a controller keeps a specific excess temperature between sensing elements at a constant level resulting in a flow dependent heater voltage. To achieve cost-effective sensors, the fabrication of the transducer is fully based on printed circuit board technology allowing low-cost mass production with different form factors. In addition, 2D-FEM simulations were carried out in order to predict the sensor characteristic of envisaged setups. The simulation enables a fast and easy way to evaluate the sensor’s behaviour in different fluids. The results of the FEM simulations are compared to measurements in real environments, proving the credibility of the model.

## 1. Introduction and Motivation

Heating, ventilation, and air conditioning (HVAC) systems are one of the most important energy consumers in modern buildings. Analyses promise that up to 40% of the energy demand can be saved through optimization of HVAC systems, other studies have come up with figures up to 60% [1,2]. The energy consumption of an HVAC system depends on the matching of individual components and how well they can be controlled to meet the requirements of the user regarding room climate and comfort.

Being able to monitor and control the energy flow in parts of an HVAC system is thus desirable both from a technical (e.g., with respect to better control) and an economical (e.g., with respect to cost savings) perspective. Essentially, this requires determining the volume flow of water and air in heating pipes and air ducts, respectively, and how they are distributed in the system. This requires a number of strategically positioned flow sensors, which in turn should be robust and cost-effective to keep the system affordable.

There are several types [3] of flow rate sensors such as differential pressure, turbine, positive displacement, Coriolis, vortex, ultrasonic, magnetic, optical, sonar, and thermal flow sensors [4,5]. Thermal flow sensors [6] are particularly appealing because of their simple construction and robust operation principle which meets the requirements of HVAC applications. There are existing thermal sensor technologies presented by Abegaz et al. [7], by Umar et al. [8] or others [9,10,11], but there is a lack of cost-effective sensors, which can measure over the whole diameter with sufficient precision. A sensor based on printed circuit board (PCB) technology was already developed, manufactured, and characterised for air ducts by the authors [12]. Moreover, this PCB-sensor was tested in water [13,14] without modification applying constant current mode operation scheme. However, the measurable flow range for both air and water was limited and not sufficient for the typical velocities in HVAC systems.

In this paper, we present an alternative approach facilitating a constant temperature mode, providing an increased performance of the sensor in terms of suitable measurement range. To achieve this, a controller keeps a specific excess temperature between sensing elements at a constant level. With increasing flow velocity, the controller adjusts the heating to maintain this excess temperature; therefore, the heating voltage becomes a function of the flow velocity. Furthermore, measurement principle and readout method permit measuring the flow velocity for various fluids. The first results have been already presented [15], and this paper extends these results.

In the following, Section 2 introduces the PCB-sensor concept for gases as well as for liquids. Section 3 presents the simulation setup with results. In Section 4 and Section 5, the experimental setups are explained in detail and measurement results are presented for both air and water environments. Finally, Section 6 and Section 7 provide a discussion, conclusions and an outlook on future work.

## 2. PCB-Sensor Design

**Figure 1:**
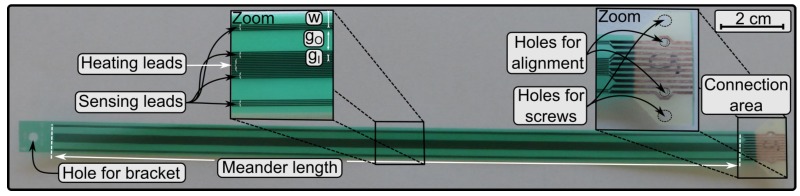
Image of the sensor layout with a meander length $l_{m}$ of 250 mm. The left inset shows the copper leads (dark stripes) in detail: In the middle there is the heater and above and below there are the four sensing leads; $g_{I}$, $g_{O}$, and *w* denote the geometrical parameters (see Table 1). The right zoom section shows the interconnection area of the sensor with holes for alignment pins and screws. The substrate is coloured beige and the solder resist is coloured green. On the left end, there is a hole for a bracket to fasten the sensor to the pipe.

The sensor geometry is depicted in Figure 1 and consists of three parts: a substrate, a conductive layer, and a cover layer. The substrate is a conventional FR4 glass epoxy laminate from Isola (Chandler, AZ 85226, USA) and exhibits a thickness of 100±13 μm. Relevant properties of this substrate are its thermal conductivity $\lambda_{\mathrm{FR}4}=0.38\;W/mK$ and its specific heat capacity $c_{P}=920\;J/kgK$.

A flexible copper foil is used as a conducting path (leads) with a thickness of 5±1.7 μm. These copper foils, on a 35 μm thick carrier foil, are fabricated from a high grade pure copper, which is dissolved in sulphuric acid ($H_{2}{\mathrm{SO}}_{4}$) forming the copper electrolyte solution. The two types of leads, one heater lead and four sensing leads, have a width of 100 μm. The heater lead, placed in the middle of the sensor, supplies the sensor system with heating power. For gases, the heater is arranged in a meander pattern with the order of eight while for liquids it is with the order of four. Besides the heater, there are the sensing leads, two upstream and two downstream, with a meander pattern with the order of four for gases and two for liquids. Other geometrical and electrical parameters (see Figure 1) are: the meander length $l_{m}$, the inner gap $g_{I}$, the outer gap $g_{O}$, the wing *w*, the heater resistance $R_{H}$, and the resistance of the temperature sensors at room temperature $R_{S}$ (see Table 1). Moreover, the copper layer has been characterized regarding its thermal thin-film properties [16] and the TCR (Temperature Coefficient of Resistance) is in accordance with the values that can be found in literature [17]. On top of the leads, a polymer layer is deposited for protection from corrosive media. It is a photostructurable kapton solder resist (polyimide film) with a thickness of 30±10 μm.

On the right side of the sensor, there are the contact pads to connect the sensor with the female PCB edge connector. This technology greatly simplifies the handling of the sensor in various experiments. Additionally, the region near the pads is equipped with holes for screws and alignment pins to position the sensor exactly with respect to the connector pins. Thereby, a reliable low resistance connection can be established. On the left end of the sensor is a hole drilled for a bracket to fasten the sensor to the flow channel.

## 3. Simulation

**Figure 2:**
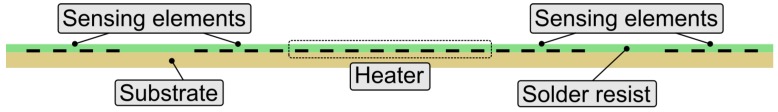
FEM model cross-section. Heating and sensing elements (copper) are placed on top of a substrate (FR4 glass epoxy), passivated by a solder resist (polymide).

A 2D-FEM (Finite Element Method) model is built with COMSOL Multiphysics (Version 5.2a, COMSOL Inc., Burlington, MA, USA) in order to predict the sensor characteristic. The applied conjugated heat transfer model couples the heat transfer module with the laminar flow module in a multiphysics simulation (nonisothermal flow). It solves the incompressible Navier–Stokes equation to obtain the velocity field inside the flow channel and around the sensor. Subsequently, the heat transfer equations, including conductive and convective heat transfer, are solved. The model relies on the cross-sectional view depicted in Figure 2 surrounded by a fluid in a flow channel.

The sensor is placed at the center of the flow channel surrounded by the fluid. A parabolic flow velocity profile (laminar and mean flow velocity) is imposed from the left-hand side into the flow channel. This assumption is based on a comparison with FV (finite volume) simulations [18]. The outlet (right side) is chosen to be a normal flow with zero pressure ($p_{o}=0\;\mathrm{Pa}$) and suppressed backflow. Additionally, the top and bottom flow channel walls, inlet, and outlet, are set to constant temperature (293.15 K), whereas the non-slip boundary condition is applied to all solid surfaces. The sensor solid surfaces are set to an initial temperature of 293.15 K. The heating power is set via the overall heat transfer rate.

**Figure 3:**
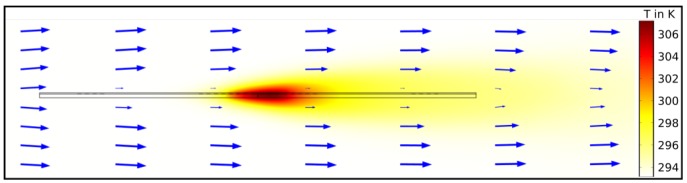
Results of the FEM simulation (for air as fluid) around the sensor. The colour indicates the temperature distribution for a heating power of 1 W while the blue arrows indicate the flow velocity field at a velocity of 1 m/s. Due to convection, the temperature profile is shifted downstream.

There are two types of fluid materials in this simulation: gases (air, argon, helium) and liquids (water, ethanol, engine oil) and for each type the geometrical parameters are adjusted accordingly (see Figure 1). For each fluid material, a stationary study with an auxiliary sweep was chosen to perform the simulation because interesting time constants are already known from previous simulations. For thermal sensors, they are rather long, here around 20 s until the settle point for accurate measurements is reached. The auxiliary sweep changes two parameters: the mean inlet flow velocity $\bar{v}$ as well as the heating power $P_{H}$ (for gases and liquids different parameter ranges as well as step sizes). The simulation results yield the excess temperature of each lead for each parameter combination and fluid material. In Figure 3, the temperature distribution (colour) and the velocity field (blue arrows) of the simulation are shown where a heating power of 1 W, a flow velocity of 1 m/s, and air as fluid are applied. It can be seen that the temperature profile is shifted downstream when a flow occurs, which means that the up- and downstream sensing elements experience different temperatures.

The results of the simulation are four sensing and one heater lead temperature matrices (for each fluid material) where the rows and columns represent the flow velocities and heating powers, respectively. From these matrices, a new matrix, the ΔT-matrix, is calculated. Subsequently, every cell of the ΔT-matrix is the sum of the inner sensing lead temperatures minus the sum of the outer sensing lead temperatures:

(1) $$\Delta T=\left(T_{\mathrm{UI}}+T_{\mathrm{DI}}\right)-\left(T_{\mathrm{UO}}+T_{\mathrm{DO}}\right),$$

where $T_{\mathrm{ij}}$ are the individual lead temperatures and the indices indicate the position on the sensor: U/D stands for up- and downstream and I/O for inner and outer position. Basically, this ΔT-matrix stores the temperature difference between the mean temperature at the middle of the sensor and the mean temperature at the periphery of the sensor for each simulated flow velocity and heating power. Then, a specific temperature difference is chosen and, from the related contour line, the corresponding matrix indices are identified. They point directly to the required heating power for this selected temperature difference at a certain mean flow velocity.

**Figure 4:**
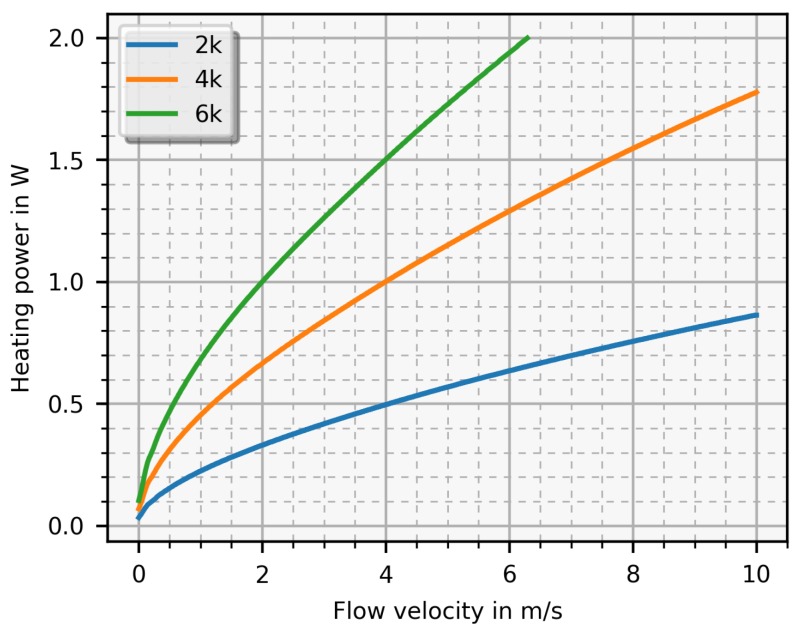
Simulated heating power versus the the flow velocity for three preselected temperature differences (ΔT) with air as test fluid.

**Figure 5:**
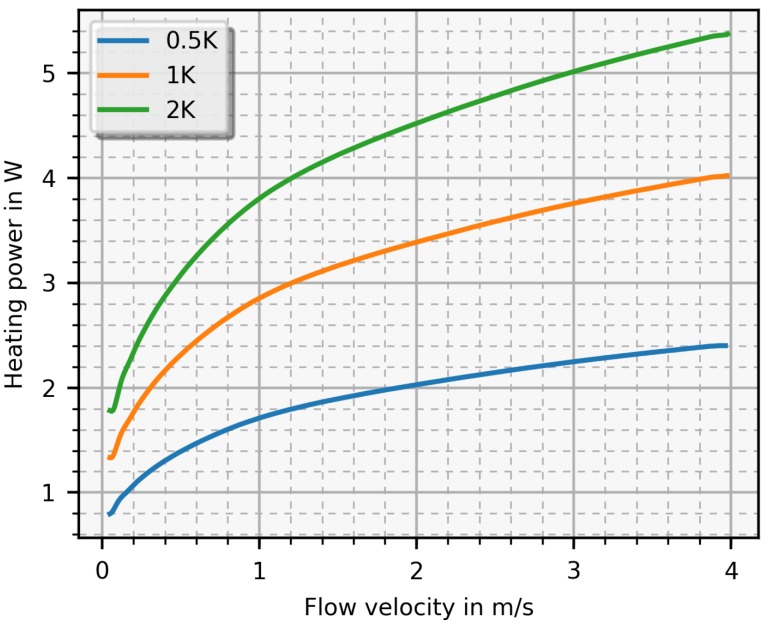
Simulated heating power versus the the flow velocity for three preselected temperature differences (ΔT) with water as test fluid.

In Figure 4, this heating power is depicted as a function of the mean flow velocity for three values of the temperature difference with air as fluid. As can be seen, this function increases monotonically with increasing flow velocity from 0 m/s to 10 m/s. Figure 5 depicts nearly the the same results as Figure 4 except the fluid is water. By changing the temperature difference ΔT, the sensitivity and measurable flow range as well as the required heating power can be adjusted. A high temperature difference allows a higher sensitivity, but, unfortunately, also requires more heating power and limits the measurable flow range. Applying too much heating power is in contrast to the targeted energy reduction and, at some point, the sensor will be destroyed when the heating power reaches a critical value causing delamination. The maximal value is specified by the PCB producer with 260 ${}^{\circ }C$ over 60 min. Therefore, a trade-off between sensitivity and heating power has to be made.

## 4. Measurements in Air

### 4.1. Setup

The constant temperature approach had to be experimentally proven, especially in higher velocity ranges. Therefore, an electronic circuit was developed which controls the heater voltage to maintain a constant temperature difference. Each sensing lead must be measured individually and they are combined according to Equation (1) after amplification. For this amplification, four instances of a high accuracy instrumentation amplifier (INA101, Burr-Brown, Texas Instruments Inc., Dallas, TX, USA)) are used as this front-end of the controller. It offers external offset adjustment via an external voltage that can be used to null its output at zero flow. This allows for adjustment at the resistance differences of the sensing leads.

**Figure 6:**
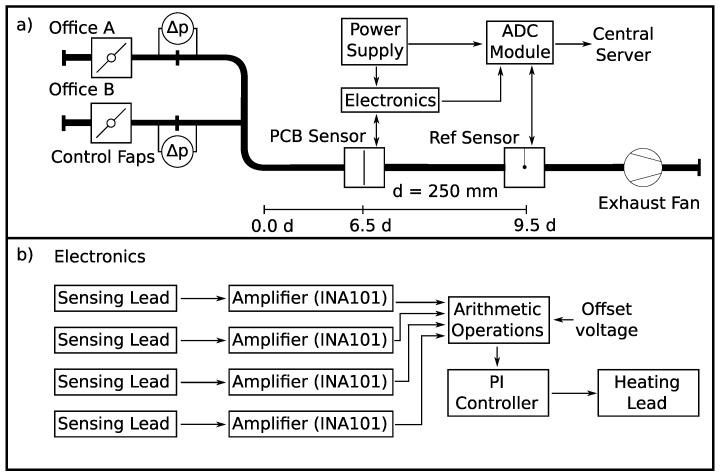
(**a**) setup in the office building with the position of both PCB and reference sensor. The flow is regulated via two flaps, differential pressure sensors (Δp), and an exhaust fan. The test flow channel is a merging pipe from two offices with a diameter (d) of 250 mm; (**b**) schematic view of the electronics. Four amplifiers evaluate the sensing lead voltages. Then, the four signals are summed according to Equation (1). The resulting signal is the command value for the PI-controller, which regulates the voltage of the heating lead to maintain the desired excess temperature (configured via the offset voltage).

Next, the choice of the controller for the heat source is explained. In addition to the PCB for conditioning of the sensing signals, an analog controller can be easily added to form a compact sensor interface. Therefore, a PI-controller was chosen over a PID-controller because the time constants of the system are rather slow as well as the D-element may tend to instability. A flexible PI-controller with separate adjustment of P and I parameters was designed. With the tuning rules from Chien, Hrones, and Reswick [19] and the step response of the controlled system, the controller parameters ($k_{r}$ and $T_{N}$) are found. These values are achieved by the parameter of passive components in the PI-controller circuit. Figure 6b depicts a schematic view of the electronics.

**Figure 7:**
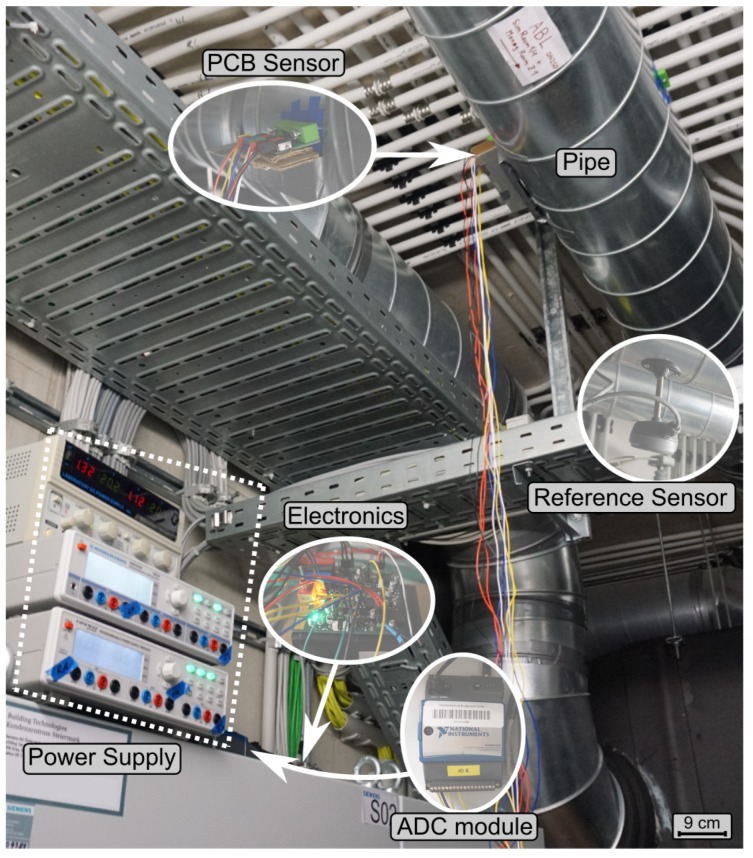
Measurement setup where the PCB sensor is mounted across a pipe. A reference flow sensor is located 750 mm downstream of the PCB sensor. The signals from both sensors are recorded with an ADC module which sends the data via WLAN to a central server.

**Figure 8:**
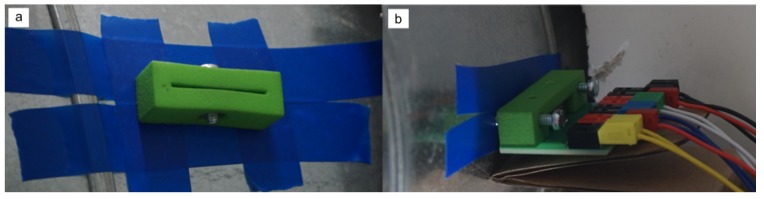
Mounting of the sensor in the flow channel via two slots. (**a**) 3D printed part (green) for tip of the sensor with a screw for tightening. (**b**) Sensor locked on the linkage PCB. The sensor is placed on a female connector and tightened via a 3D printed part (green).

The experiments were performed in ventilation pipes of a living-lab office building [18]. Figure 6a depicts the measurement setup as well as a sketch of the ventilation system. The PCB sensor was installed into a straight (Figure 7), DN250 spiral air duct section, 6.5 diameters (d=250 mm) downstream a 90${}^{\circ }$ bend. Although the space was restricted, installation was easy and fast. Only two holes had to be drilled, which is a common way to install sensors into air ducts. The sensor was spanned across the pipe with two mounts attached to the pipe to hold the sensor (Figure 8).

A reference sensor (Produal IVL 10 hot wire anemometer, Produal Oy, 48770 KOTKA, Finland) was located 750 mm downstream of the PCB sensor with a stated absolute accuracy of ±0.5 m/s + 7 % from reading. The PI-controller, power supply, and ADC (analog digital converter) transmitter module were placed near the two measuring devices. This ADC transmitter module was an NI WSN-3226 node (National Instruments, 1010 Vienna, Austria) and recorded the values from the PI-controller and the reference sensor. The flow is exhausted by a central fan unit from two offices A and B where control flaps ensure the defined flow rates. In detail, the desired flow rates are established with the pre-installed volume rate meters at each control flap, accessed by the central building automation system. This volume rate meters are membrane differential pressure transducers (Figure 6 Δp) from Siemens (GDB181E/3) (Siemens AG Austria, 1210 Vienna, Austria) with a declared absolute accuracy of ±7.5 Pa. Therefore, it was possible to manually regulate each controller, so that the air flow velocity could be set at well-defined rates ranging from low (exhaust only from one room) to high velocities (exhaust from both offices).

### 4.2. Results

Before the measurements could be performed, the operating point of the sensor must be defined. Therefore, all four sensing lead currents are set to 10 mA so negligible self-heating is assured which has been tested in previous work. At zero flow velocity and without heating, the output signal of each front-end is set to 0.5 V. Next, the set point voltage which determines the excess temperature was adjusted to 55 mV (corresponds to ΔT=5 K) leading to a heater voltage of 6.6 V at zero flow velocity. This is necessary because the system cannot be cooled actively, only passively by turning off heating. However, if the PI-controller receives a negative command value, the sign of controller output current changes, but this does not mean that the heater absorbs energy. Therefore, these offset values are used to avoid a negative command value.

**Figure 9:**
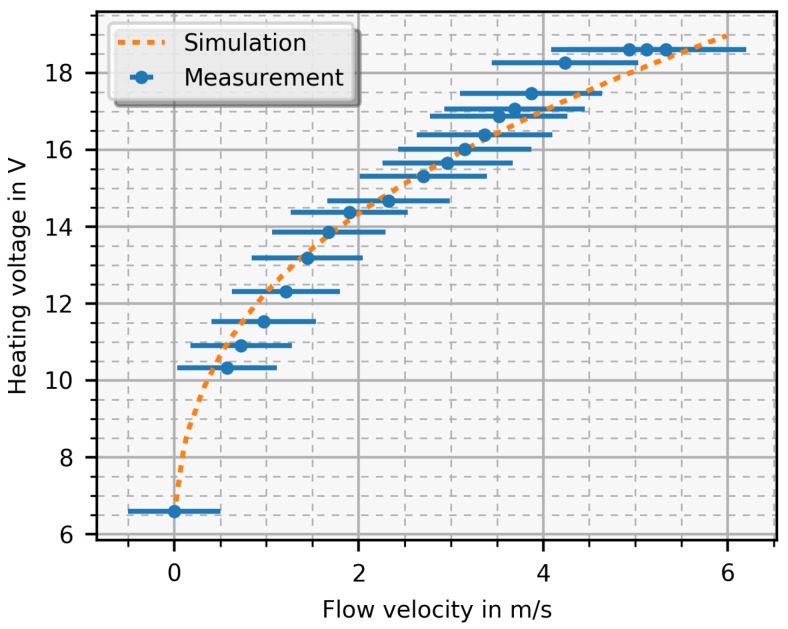
Measurement of the PCB sensor in constant temperature mode compared with the simulation. The heater voltage is plotted as a function of the mean flow velocity where the error bars indicate the error (±0.5 m/s + 7% from reading).

In Figure 9, a measurement result is depicted as well as a simulation result for comparison where the heater voltage is plotted as a function of the mean flow velocity. The error bars indicate the accuracy of the reference sensor. As the simulation predicted, the function increases monotonically with increasing flow velocity and, with this setup, flow velocities from 0.5 m/s up to 5 m/s are possible. For the presented simulation results, the heater voltage was calculated from the required heating power gained from the difference temperature matrix. Thereby, a 5 K temperature difference was chosen and, with the 6.6 V offset voltage, the simulation result is in good agreement with the measurement.

In practice, the heating voltage is measured and the flow velocity is the desired quantity. Therefore, heating voltage and flow velocity of the simulation (see Figure 4 with 5 K temperature difference) are switched and a function $f_{\mathrm{Fit}}\propto$ Flow (unit is m/sI) was then fitted with a nonlinear least squares fit. With this function, it is possible to transform the measured heating voltage $V_{h}$ into the mean flow velocity and it is expressed as:

(2) $$f_{\mathrm{Fit}}=0.0002\;\cdot \;{\left(V_{h}\right)}^{3.499}-0.157.\;$$

This equation proves the expected relation between heating voltage (corresponds to the heating power with the power of two) and flow velocity, known as flat plate heat transfer theory [20].

**Figure 10:**
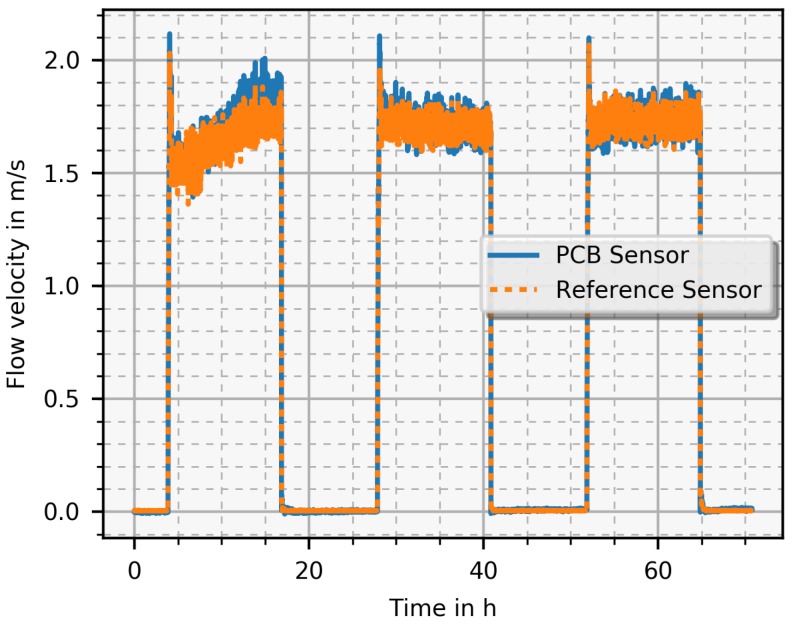
Long-term measurement where the sensor was tested in a normal operation mode of the HVAC system that was controlled by an automatic system. PCB and reference sensor were measured every 10 s during three days. The scaling factor for the PCB sensor is given in Equation (2).

Another measurement focused on long term behaviour of the PCB sensor (Figure 10) to characterise the drift under real operating conditions. Therefore, the PCB and the reference sensor measured the flow velocity in a normal working mode over a couple of days. This requires a control system that adjusts the outlets and ventilator speeds automatically to the programmed values. In the evening, the system shuts down, leading to no air flow. In the morning, the system starts and keeps the temperature and fresh air flow at the defined level over the working period into the afternoon. Both PCB and reference sensor exhibit the same behaviour, which proves that the PCB sensor was working correctly over three days. The scaling factor for the PCB sensor is given in Equation (2). A major result is that there was no measurable drift of the zero point during this time period.

**Figure 11:**
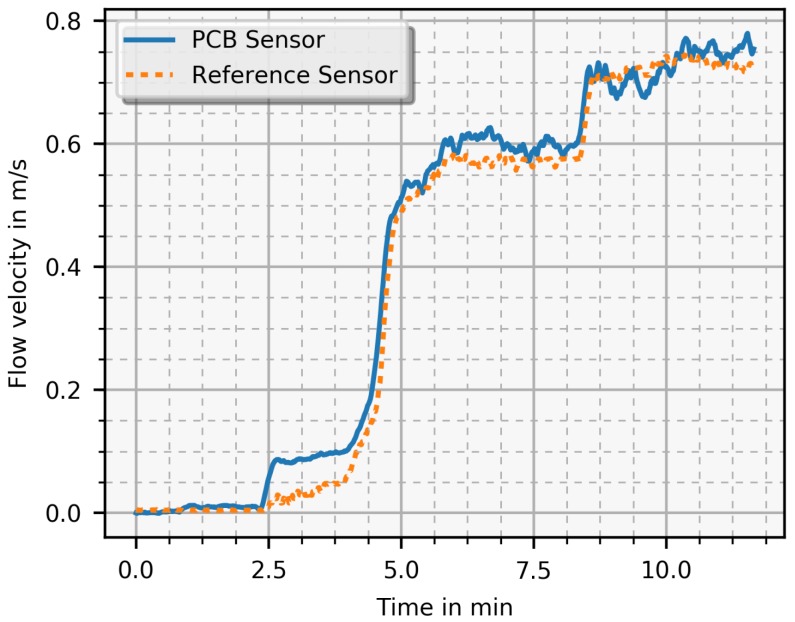
Start-up detail (timely zoomed in) of a measurement (similar to Figure 10). The PCB sensor recognises small flow velocities (at 2.5 min) before the reference does. Both sensors need the same settling time before an accurate value is achieved. Detailed information about the response property can be found here [12].

A closer look at the start-up of a measurement revealed a higher sensitivity of the PCB sensor where the flow velocity was slowly increased in small steps. In Figure 11, a detailed section of a measurement is shown where the PCB sensor can detect smaller flow velocities than the reference sensor at the same settle time. However, due to the limited accuracy of the reference sensor (±0.5 m/s + 7% from reading), the PCB sensor cannot be calibrated properly, allowing only for qualitative evaluation of the PCB sensor. This stresses the urgent need for a specifically designed, robust, and cost-effective alternative flow velocity sensor.

## 5. Measurements in Water

### 5.1. Setup

**Figure 12:**
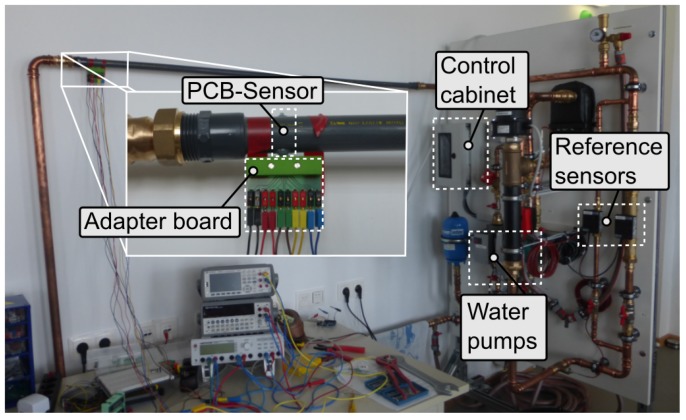
Picture of the water experimental setup. The sensor is stretched across a PVC water pipe (inner diameter of 21.2 mm), located at 135 cm at a strait pipe which is 20 cm before a bend, and connected to the controller by an adapter board. The volumetric flow is controlled via two separate water pumps and measured with two ultrasonic flow sensors. From a control cabinet, all sensors and actuators are controlled.

The experimental setup for water measurements is illustrated in Figure 12. It consists of a water circuit system with pumps and reference sensors, a control cabinet, the test flow channel with the PCB-sensor, and its equipment for readout. A PVC-U pipe with a total length of 145 cm and an inner diameter of 21.2 mm is used as test flow channel. The installed pumps allow a volumetric flow in the range of 1.3 $m^{3}$/h to 4.1 $m^{3}$/h, which means, for the test pipe, mean flow velocities from 0.15 m/s to 3.22 m/s. Additionally, the temperature can be controlled with an electric heating element in combination with a continuous water cooler. This setup allows for steering fluid temperatures from 20 ${}^{\circ }C$ to 90 ${}^{\circ }C$. Two ultrasonic flow sensors and two Pt100 temperature sensors are installed as reference sensors for fluid velocity and fluid temperature, respectively. All industrial actuators and sensors are controlled from a central control cabinet hosting a Raspberry Pi with an Arduino shield, the power-supply, and several electronic amplifiers. Via a python script, the desired velocity and temperature are set and all sensor values are logged. The PCB-sensor was situated in the PVC-U pipe and connected to the PI controller by an adapter board (PCB edge connector).

### 5.2. Results

**Figure 13:**
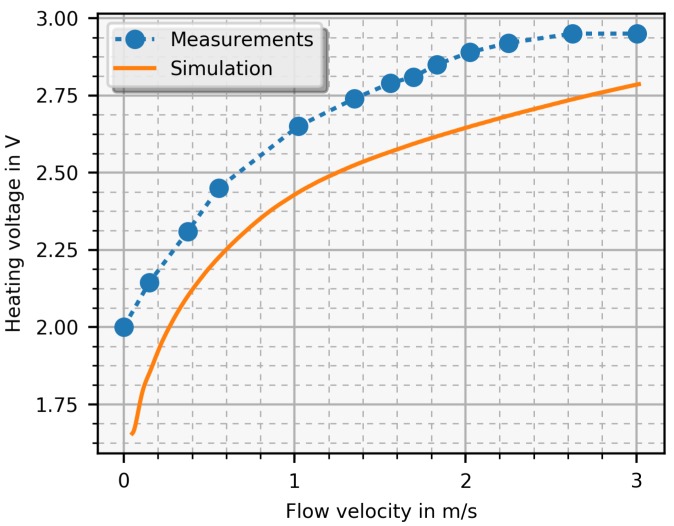
Simulated and measured heater voltage depending on the flow velocity in constant temperature mode where the temperature difference was set to 0.5 K.

The PCB-sensor with the electronics was designed to work properly in air environments. The designed amplifier stage, placed on the electronics, could not provide the needed current for higher velocities (> 2 m/s). Therefore, this amplifier stage was replaced with an audio amplifier. Basically, the audio amplifier is a voltage regulated current source and its voltage is controlled by the PI-controller. Figure 13 shows a result of experiments where the heater voltage is plotted versus the flow velocity as well as the corresponding simulation result for comparison. The voltage difference is set such that Δ*T* amounts to 0.5 K and the measured function is monotonously increasing up to 2.6 m/s until saturation is reached. The measured voltage is higher than the simulated due to several simplifications discussed in the next section (Section 6).

## 6. Discussion

Qualitatively, the simulation-based predictions of the sensor behaviour and the experimental results shown in the previous section match fairly well, especially with respect to the transfer characteristic. The constant temperature mode allows for a wider dynamic range than the straightforward, non-feedback controlled mode, and it is applicable in air and water alike, albeit with differences regarding sensitivity and range. As expected, gaseous fluids permit a higher temperature difference of the sensing elements and therefore also a larger velocity range to be measured, up to 6 m/s are measurable while with the constant current mode only flow velocities up to 3 m/s are detectable. In liquids, saturation is reached at much lower velocities, around 2.6 m/s (<1 m/s in constant current mode) because of their higher thermal conductivity, which leads to a higher heat dissipation.

According to the thermal properties of the medium, the heating power needed to achieve the desired temperature difference (e.g., 2 K) is higher for water than for air (at 2 m/s the heating power for water is 4.3 W while for air it is 0.3 W). First and foremost, the thermal capacity of liquids is much higher than that of gases, which means that more energy is needed to heat up an aqueous fluid, e.g., for a ΔT=2 K and flow of 2 m/s there is a factor of 14 between water and air. This introduces a hard limit of the measurement range because at some critical current density, the PCB-sensor will get thermally damaged or even destroyed. This critical value, found by increasing the heating power in an air duct until the sensor was destroyed, amounts to 2000 A/mm^2^ for air while, for water, the critical current density is calculated between 4000 A/mm^2^ to 6000 A/mm^2^. To prevent thermal destruction, the temperature difference was set to 0.5 K enabling a maximum detectable flow velocity of 2.6 m/s.

The comparison between simulation and measurement results reveals a noticeable difference, as shown in Figure 12. What causes this difference? The primary reason is that the simulation is based on several simplifications. Firstly, it applies a 2D FEM model which assumes that the temperature field does not change perpendicular to the flow direction. In reality, the lateral extension is finite with decreasing velocity towards the pipe or duct walls. In the simulations, this effect was partly compensated for by calculating with a mean velocity value instead of the maximum predicted by the 2D model. Another limitation is the assumption of a laminar flow. This was done for simplicity in order to demonstrate the basic function of the sensor. In reality, this assumption holds only for low velocities, whereas, towards the upper end of the measurement range, the flows become turbulent. The profile of a turbulent flow in a straight pipe reasonably far away from bends, however, is flatter across the diameter than the parabolic laminar profile, which actually supports the simplification of the 2D model. Furthermore, the sensor has an averaging effect in both spatial (across the diameter) and temporal respect (because of its thermal inertia, which levels out fluctuations in a turbulent flow). Another small influence comes from geometrical details of the sensor. The reversing loops of the leads at the tip of the sensor as well as the connection lines at the bottom of the sensor distort the temperature field. A small amount of injected heat power flows via the PCB and leads directly to the environment without contribution to the convection process. Moreover, there are supply lines to the heater and contact resistances, which are not modelled in the simulation. Taking all these effects together, more heating power is needed to operate the sensor at a given temperature. In addition, saturation is reached faster in reality than in the simulation.

## 7. Conclusions and Outlook

We presented a thermal flow sensor in constant temperature mode for various fluids. While the feasibility of PCB-based calorimetric flow sensing, applying constant current mode, has already been demonstrated [12,13], this paper investigated the applicability of such a PCB-sensor in constant temperature mode in various environments. Extensive simulations, based on a 2D FEM model, for different fluids have been carried out, followed by new measurement results for both air and water setups. Moreover, the sensor in constant temperature mode was tested in a real HVAC environment and compared to a commercial sensor.

While the accuracy of the reference sensor was limited (±0.5 m/s + 7% from reading), flow velocity and long-term measurements were performed and compared to simulation results to prove the credibility of our modelling approach. Next, the PCB-sensor was tested in a water pipe. To implement the constant temperature mode, a closed loop operation with an electronic controller was set up. The results essentially confirmed the simulation and demonstrated the basic functionality of the device.

Future work will focus on optimising the design of the PCB sensor for aqueous fluids. In order to achieve a more efficient sensor, the transducer design will be adapted: when the inner sensing leads are located closer to the heater (<100 μm), less heating power is required to heat up the sensing leads. The interconnection to the sensing leads should be enlarged and redesigned to prevent a possible overheating in the channel wall. It should be ensured that the small sensing leads are only in contact with the liquid and nothing else.

In the present design, a flexible PCB was used for the sensor, whereas a rigid PCB was used for the front-end electronics. It would be beneficial to place both functional parts on one PCB. The electronics could be placed onto the flexible PCB, or a semiflex PCB could be used. The flexible part of the semiflex PCB could act as a sensor and the rigid part could carry the electronic components. By augmenting the sensor with a microcontroller with wireless communication interface, the sensors could be connected to a wireless sensor network and a gateway to, e.g., a facility management system.

The appealing feature of these PCB-based sensors is that they can be easily adapted to any pipe dimensions and installed very quickly in gaseous as well as in aqueous environments. They are able to measure flow accurately enough to allow the detection of inefficiency in an HVAC system while still being cost-effective and robust. Ultimately, they can serve to recalibrate HVAC systems in existing buildings to work more energy efficiently, which will contribute to the goal of reducing overall energy consumption.

## Figures and Tables

**Table 1:** Geometrical and electrical parameters of the sensor: $l_{m}$ is the meander length of the leads, $g_{I}$ denote the gap between the heater and the inner sensing leads, $g_{O}$ the gap between the inner and outer sensing leads, and *w* specifies the distance (wing width) between the outmost leads and the PCB edges. $R_{H}$ denote the heater resistance and $R_{S}$ the mean resistance with standard deviation of the sensor.

Media	$l_{m}$	$g_{I}$	$g_{O}$	*w*	$R_{H}$	$R_{S}$
Gas	250 mm	0.1 mm	2.5 mm	1 mm	163 Ω	85±6 Ω
Liquid	30 mm	0.1 mm	0.1 mm	0.2 mm	6.7 Ω	3.8±0.1 Ω
